# Insights Into Genome-Wide Association Study for Diabetes: A Bibliometric and Visual Analysis From 2001 to 2021

**DOI:** 10.3389/fendo.2022.817620

**Published:** 2022-03-11

**Authors:** Yang Liu, Yun Wang, Shan Qin, Xinye Jin, Lingzi Jin, Weijun Gu, Yiming Mu

**Affiliations:** ^1^ Department of Endocrinology, the First Medical Center of Chinese People’s Liberation Army (PLA) General Hospital, Beijing, China; ^2^ Department of Endocrinology, the Eighth Medical Center of People’s Liberation Army (PLA) General Hospital, Beijing, China; ^3^ Department of Nephrology, Hainan Hospital of Chinese People’s Liberation Army (PLA) General Hospital, Academician Chen Xiangmei of Hainan Province Kidney Diseases Research Team Innovation Center, Sanya, China; ^4^ Department of International Medical Services, Peking Union Medical College Hospital, Beijing, China

**Keywords:** diabetes, bibliometric analysis, visualization, CiteSpace, VOSviewer, genome-wide association studies

## Abstract

Hundreds of research and review articles concerning genome-wide association study (GWAS) in diabetes have been published in the last two decades. We aimed to evaluate the hotspots and future trends in GWAS in diabetes research through bibliometric analysis. Accordingly, 567 research and review articles published between 2001 and 2021 were included. A rising trend was noted in the annual number of publications and citations on GWAS in diabetes during this period. Harvard University and Harvard Medical School have played leading roles in genome research. Hotspot analyses indicated that DNA methylation and genetic variation, especially in type 2 diabetes mellitus, are likely to remain the research hotspots. Moreover, the identification of genetic phenotypes associated with adiposity, metabolic memory, pancreatic islet, and inflammation is the leading trend in this research field. Through this review, we provide predictions on the main research trends in the future so as to shed light on new directions and ideas for further investigations on the genetic etiology of diabetes for its prevention and treatment.

## Introduction

The 9th edition of the International Diabetes Federation’s Diabetes Atlas has estimated that, presently, 9.3% of adults (463 million people) are living with diabetes (1). Type 2 diabetes mellitus (T2DM) is the most common type of diabetes with a strong genetic predisposition. Compared with individuals without parental diabetes, the odds ratios (95% confidence interval) for the prevalence of T2DM in those with maternal, paternal, or bilineal diabetes were 3.4 (2.3–4.9), 3.5 (2.3–5.2), and 6.1 (2.9–13.0), respectively (2). Progress in genome-wide association study (GWAS) and global collaborations in the field of genome projects have enabled the identification of 243 new candidate loci associated with susceptibility to T2DM from 32 European-descent GWAS, including 74,124 T2DM cases (3). Furthermore, 61 loci newly implicated in T2DM predisposition have been identified in the East Asian population (4). Another noteworthy progress is the discovery of 318 novel risk loci linking T2DM susceptibility to diverse biological processes in a large-scale multi-ethnic GWAS encompassing over 1.4 million participants (5). However, the common variants explained only a small proportion (10%–15%) of this variance in T2DM risk (6). Moreover, the location of most variants in the intergenic or the intronic region and the presence of linkage disequilibrium (7) pose a challenge in deciphering their function. Thus, we inferred that the causal relationships and the mechanisms by which these variants exerted their effect on the pathogenesis of T2DM require in-depth investigation.

Bibliometric analysis is a convenient and accurate tool for discovering the hotspot and trend in a specific research field. Summarizing the number of publications produced by various countries/regions, institutions, and authors can shortlist the leading researchers in the field to equip future researchers with more valuable references for further investigations. Meanwhile, bibliometrics assists the prediction of developments and trends in diseases (8). No bibliometric study has so far been performed in the field of GWAS in diabetes (9). Thus, in the present study, comprehensive visualization and bibliometric analysis of genomic research in diabetes over the last two decades were performed to identify the hotspots and prospective trends in GWAS in diabetes over the next decades through co-occurrence and co-citation analysis with clustering visualization.

## Materials and Methods

### Data Sources and Search Strategy

All data employed in the present study were retrieved from the Web of Science, which is one of the most influential databases of scientific literature. We applied the Web of Science Core Collection (WoSCC) using the following search query string: (#1) TS = “genome-wide analysis” or “genome-wide association study” or “whole-genome analysis”; (#2) TS = “diabetes mellitus” or “diabetes” or “hyperglycemia”; (#3) = (#1) AND (#2). We limited the literature type to “article or review” and the language to “English.” The publication years ranged from 2001 to 2021.

### Data Collection and Filtration

The process of literature retrieval and data download was conducted on November 2, 2021. The retrieval query returned 590 results. Two investigators independently screened the title, abstract, and the set of keywords or full text to determine the correlation between the retrieval data and genetics in diabetes. Eventually, 567 results that met the significant consistency as an agreement on 0.95 were retained after effective comparison and discussion (10).

### Literature Visual Analysis

CiteSpace v5.7.R5 (Chaomei Chen, Drexel University, Philadelphia, PA, USA) was used to generate a visualization map to identify annual or cumulative publications and co-cited authors/references and to capture keywords with strong citation bursts. The time trends of keywords and the overlay visualization maps were also analyzed by CiteSpace.

The betweenness centrality firstly introduced by Freeman has been adopted to reflect the impact of a specific node in a network (11). To test the effect of the network, the modularity *Q* and the mean silhouette scores (12) served as two important metrics indicating the overall structural properties. The equation of these metrics is shown in the *Appendix*.

The VOSviewer v1.6.16 (Leiden University, Leiden, Netherlands) was adopted to visualize the intensity of cooperation between countries/regions and institutions to examine the scientific strength and international influence of these countries/regions and institutions in the field of diabetes GWAS research (13). The document co-citation network with citation burst was conducted to reveal the most concerned topics along the time. The keyword co-occurrence network visualization is a method for determining research hotspots and predicting research trends (14). Furthermore, investigation on keyword bursts added richer interpretations to the understanding of the emerging trends in the field of GWAS in diabetes rather than only considering the cumulative number of keyword occurrences (15).

## Result

### Trends in Annual Publications

**Figure 1:**
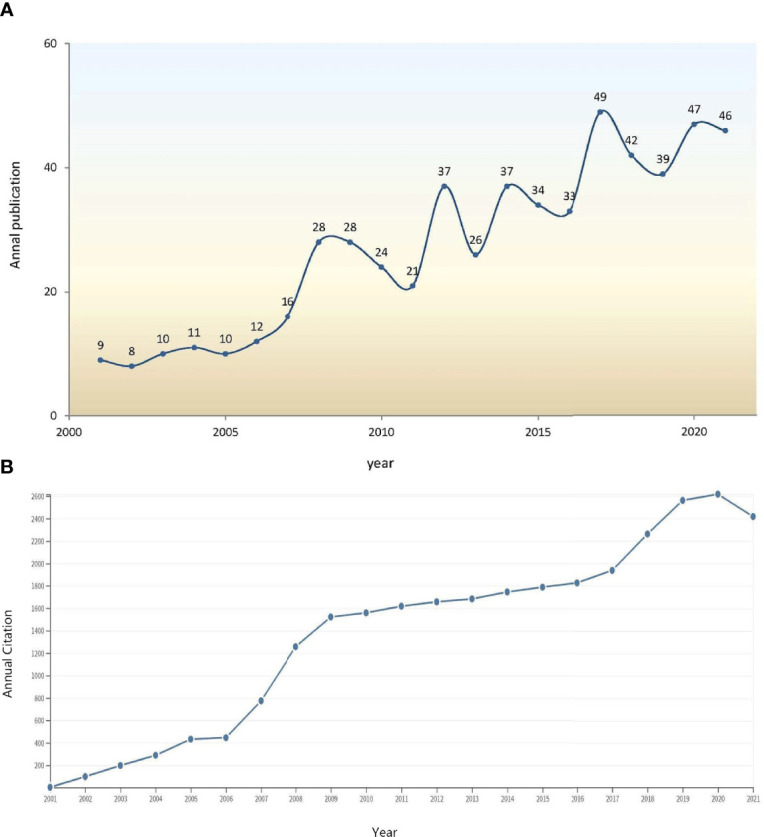
**(A)** Annual publications of GWAS research in diabetes from 2001 to 2021. **(B)** Trends in annual citation frequency of the 567 retrieved articles from 2001 to 2021.

A total of 590 papers were identified, and 567 papers (478 articles and 89 reviews) from 2001 to 2021 were eventually included. Among them, 208 (36.68%) publications were related to T2DM, and 75 (13.23%) papers focused on type 1 diabetes mellitus (T1DM). A rising trend was detected in both annual publications (**Figure 1A**) and annual citations (**Figure 1B**) associated with GWAS in diabetes. According to the WoSCC database, the 567 papers were cited 28,721 times, with an average citation frequency of 50.65 for each paper. Owing to a burst of publications since 2007 (the growth rates of the citation frequency in 2007 and 2006 were 73.93% and 3.00%, respectively), the citation frequency grew rapidly from 2007 to 2009.

**Figure 2:**
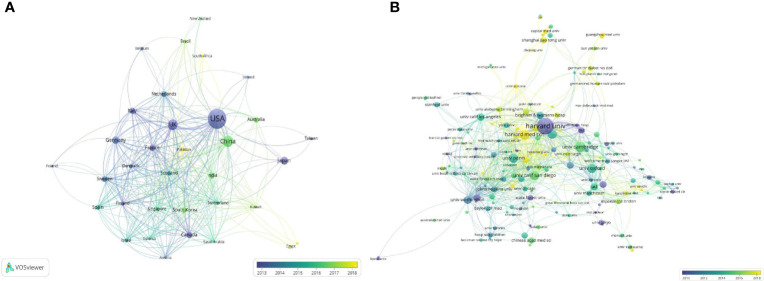
**(A)** The network of cooperation between countries/regions. **(B)** The network of cooperation between institutions based on VOSviewer. Node size indicates the number of publications; the link size refers to the cooperation Intensity; the earlier contributions were presented in darker colors.

### Contribution of Countries/Regions and Institutions

A total of 64 countries/regions contributed to publications on GWAS in diabetes between 2001 and 2021. The top 10 most productive countries/regions are listed in **Table 1**, which shows that the United States is the most productive country, with 266 articles published so far and over 17 publications annually since 2015, followed by China and the United Kingdom, with 88 and 76 published papers, respectively. Tight network connectivity was observed among these countries/regions, as indicated in **Figure 2A**.

**Table 1:** Top 10 countries/regions contributing to publications on genome-wide association study (GWAS) in diabetes.

Rank	Country/region	No. of publications	Proportion, *N* = 567 (%)	Citations per document
1	USA	266	46.91	77.94
2	China	87	15.34	20.73
3	UK	76	13.40	57.04
4	Japan	42	7.40	22.78
5	Germany	40	7.05	73.95
6	Canada	29	5.11	60.34
7	France	28	4.93	104.52
8	Italy	26	4.58	113.96
9	Sweden	25	4.40	153.56
10	Netherlands	25	4.40	84.24

The institution cooperation network illustrated in **Figure 2B** involved 460 institutions and 1,122 links. A cooperation network centered on Harvard University (with 25 publications) and Harvard Medical School (with 17 publications) was formed along with other organizations at home and abroad, suggesting a close and continuous academic cooperation among the institutions in this field.

### Document Co-Citation Network

**Figure 3:**
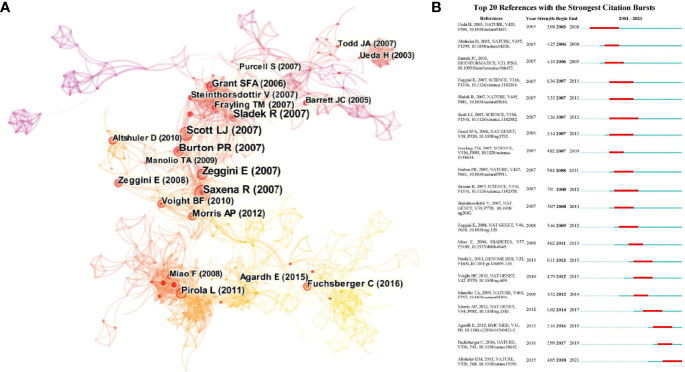
**(A)** The document co-citation network clustering. The nodes and links are distinguished by colors, in which cool-toned color refers to an earlier co-citation relationship. References with > 10 citations are displayed in the landscape in nodes named by first author (publication year). The size of the node represents the citation number. Nodes with red ring serve as the references with citation bursts meaning emergence of new trends. The links refer to the beginning of the connections. **(B)** The top 20 references with the strongest bursts. The red bar refers to the burst duration. The appearance of dark blue bar represents the publication of the article. The burst strength indicates the importance of this article to the research field.

The citation patterns of the references revealed insights into the structure and dynamics of scientific paradigms. **Figure 3A** provides a visualization of the document co-citation landscape. The synthesized network contained 805 references and 2,257 co-citation links. The three largest connected components included 481 references, accounting for 59% of the entire network. The nodes and links are distinguished by colors, in which a cool-toned color refers to an earlier co-citation relationship. References with ≥10 citations are displayed in the landscape. Meanwhile, we included the citation burst in **Figure 3B** to identify the major milestones in the development of GWAS in diabetes steering the research trends. The major milestones in the development of GWAS in diabetes could be identified as references with a high burst strength in **Figure 3B**.

### Author Co-Citation Network

**Figure 4:**
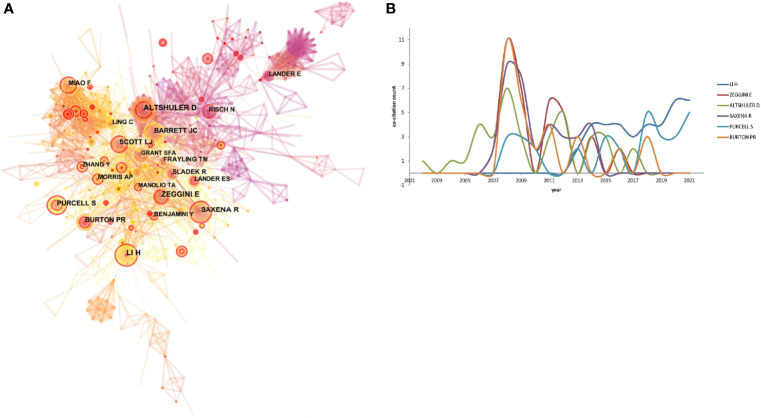
**(A)** The author co-citation network. The configurations set as: pruning pathfinder, algorithm log-likelihood rate (LLR), time slice l, top N 50 per year, link-retaining factor (LRF) = 3, look back years (LBY) = 5, and e l = 1. The node size referred to the number of citations of a specific author, while the links represented the frequency of the co-citation for two authors. The higher betweenness centrality (betweenness centrality > 0.l) represented the leading researchers and was expressed as a purple circle around the node. **(B)** The top 6 co-cited author with annul citation counts.

The author co-citation network, identifying frequently cited scholars with globally recognized publications in GWAS in diabetes, was processed by CiteSpace, shown in **Figure 4A**, achieving a network with 788 nodes and 2,218 links. The top 6 highly cited authors and their citation distributions during 2001–2021 are shown in **Figure 4B**. H. Li, E. Zeggini, D. Altshuler, R. Saxena, S. Purcell, and P.R. Burton constituted over 30 co-citations assisting the development of GWAS in diabetes in fields ranging from bioinformatics, genetic mapping to the pathophysiology of diabetes and explanations of the phenotypes.

### Analysis of Research Hotspots

**Figure 5:**
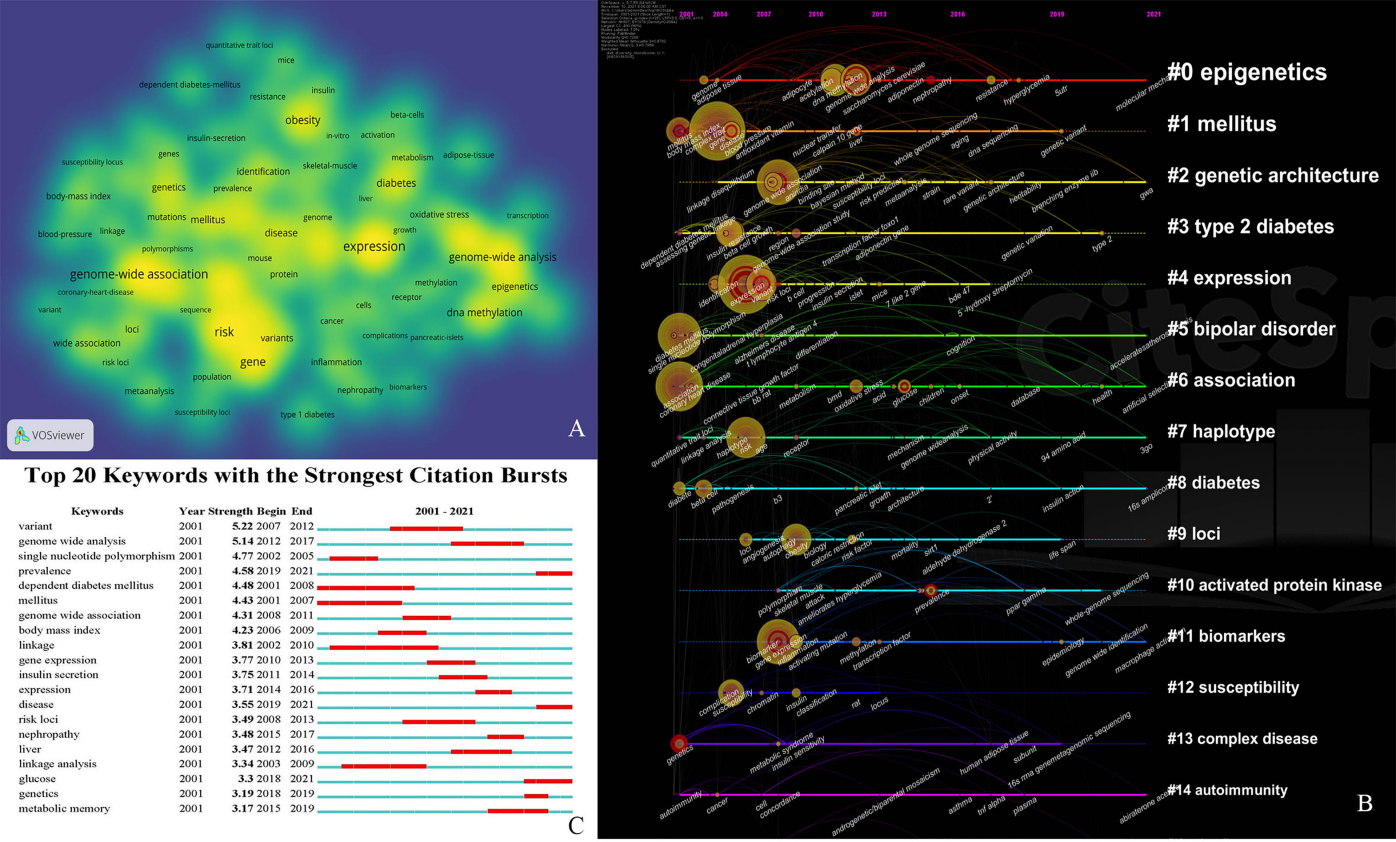
**(A)** The co-occurrence network of the most frequently occurred 40 keywords in field of GWAS in diabetes. Node size refers to number of frequency. **(B)** Timeline view of cluster for keywords co-occurrence in pathfinder pruning. Each cluster is named by most frequently occurred keyword. The clusters were arranged vertically in the descending order of their size (the smallest number refers to the largest cluster). The position and size of the node on the timeline reveal the cumulative frequency, and the year for the first occurrence of each keyword, respectively. Nodes with red tree rings refer to keywords with citation bursts. **(C)** Top 20 keywords with the strongest citation bursts. The red bar refers to the burst duration. The burst strength indicates the importance of the keyword to the research field.

The 40 most frequently occurring keywords in the field of GWAS in diabetes are presented in the VOSviewer in **Figure 5A** to visualize the emerging trends in the field. A timeline view of hotspot clustering based on keyword co-occurrence was adopted to identify the hotspot along the time, as displayed in **Figure 5B**. The cluster network consisted of 507 nodes and 2,762 links, with modularity *Q* = 0.7268, which indicated that the network was significant and convincing. The mean silhouette score was 0.8792 (close to 1), suggesting a reliable network with homogeneity among the clusters. The three largest clusters contained 490 nodes and comprised 96% of the network. Fifteen clusters (#0–14) were named by the keyword with the highest frequency in the cluster. Cluster #0, named *epigenetics*, was the largest cluster, comprising 53 keywords ranging from 2002 to 2021, with 2012 as the median year for the occurrence of this cluster. The silhouette value 0.803 was considered as indicating a relatively high homogeneity inside the cluster (detailed in **Supplementary Table S1**). The 20 most frequently occurring keywords, with the exclusion of the retrieved terms from WoSCC (shown in **Figure 5A**), based on the keyword co-occurrence clustering (shown in **Table 2**) indicated that *risk* (in cluster #3) was the most representative keyword in the first 10 years (2001–2009) of the domain, followed by *insulin resistance* (in cluster #3), which is a pathophysiological process that precedes diabetes. The identification and extraction of specific risk factors, including *adipose tissue* (cluster #0, betweenness centrality = 0.14), *obesity* (cluster #6, betweenness centrality = 0.06), *inflammation* (cluster #10, betweenness centrality = 0.09), and *body mass index* (cluster #1, betweenness centrality = 0.08), was expected to raise concerns among the researchers in such domains. The frequently employed keywords in the recent decade, such as *epigenetics* (top 1 in cluster #0, betweenness centrality = 0.04, appeared in 2011), *DNA methylation* (top 2 in cluster #0, betweenness centrality = 0.05, appeared in 2011), *metabolic memory* (cluster #0, betweenness centrality = 0.02, appeared in 2012), *pancreatic islet* (cluster #3, betweenness centrality = 0.02, appeared in 2012), and *oxidative stress* (cluster #14, betweenness centrality = 0.00, appeared in 2012), indicated the recent focus on the in-depth cellular and molecular mechanisms, especially the epigenetic aspect, in the prevalence of diabetes.

**Table 2:** The 20 most frequently occurring keywords for genome-wide association study (GWAS) in diabetes research (derived from the list in **Figure 5A**), excluding the terms retrieved from the Web of Science Core Collection (WoSCC).

Frequency	Keywords	Year of first appearance	Centrality
54	Risk	2006	0.02
37	Insulin resistance	2005	0.08
35	DNA methylation	2011	0.05
35	Obesity	2009	0.06
29	Susceptibility	2005	0.05
25	Epigenetics	2011	0.04
20	Beta cell	2003	0.06
19	Inflammation	2009	0.09
19	Adipose tissue	2004	0.14
18	Body mass index	2002	0.08
15	Protein	2008	0.04
15	Cell	2007	0.06
13	Glucose	2014	0.09
13	Meta-analysis	2014	0.03
13	Oxidative stress	2012	0
13	Metabolic memory	2012	0.02
13	Metabolism	2009	0.03
12	Prevalence	2015	0.03
11	Pancreatic islet	2012	0.02
11	Risk factor	2012	0.02

Keyword burst analysis revealed the top 20 emergent keywords with the strongest citation bursts, as shown in **Figure 5C**. The keywords were ranked based on the strength of bursts. *Variant* (in cluster #4) and *genome-wide analysis* (in cluster #5) received the highest bursting attention across the entire span. In the early years, investigating the genetic basis of *insulin-dependent diabetes mellitus* (in cluster #3) was one of the most important goals. Recently, there has been a growing interest in diabetes-related complications, including *nephropathy* (in cluster #0), and novel mechanisms in the development of the disease, such as *metabolic memory* (in cluster #0).

## Discussion

A rising trend in publications and citations on GWAS in diabetes was observed during 2001–2021, which suggests that it remains an area of concern. With regard to the contribution of countries/regions, the publications and citations per document (77.94) in the United States have obvious advantages over other countries/regions, which signifies the country’s scientific research strength and high investment in the field. China ranks second in the number of papers published, but the citations per document were only 20.73, which signifies the need to improve the quality of research. To assess the intensity of cooperation among the nations in the field over time, dual-map overlays were constructed, which showed that the North American and European countries/regions made their contributions in the early years, i.e., before 2014. In 2016, there was a burst in publications from China, which increased from 8 publications in 2016 to 13 publications in 2021. Furthermore, there was a close connection among countries/regions, and the United States has been the most frequently involved country in international cooperation. In the distribution of contributions among institutes, Harvard University was an early contributor to genome research in diabetes, and its contributions began way back in 2001. The most cited article was published in 2007 by Saxena et al., affiliated with Harvard University. In this research, the investigators analyzed 386,731 common single-nucleotide polymorphisms to identify three loci associated with T2DM (16). This has been by far the most cited article, with a total of 2,178 citations. Harvard Medical School had a burst of publications in 2016. Fuchsberger et al., from Harvard Medical School, investigated the genetic architecture in T2DM and concluded that large-scale sequencing is necessary to identify functional alleles that provide important clues to complex disease pathophysiology. Furthermore, the researchers mentioned that common variants seem to be the predominant contributors to T2DM heritability. Their work has been cited 597 times, ranking the first in the recent 5 years (5th in 20 years from 2001 to 2021) (17).

The top 20 references with the strongest bursts referred to knowledge transition and the emergence of new trends in the research field. The first milestone paper in the field was on the identification of polymorphisms in the cytotoxic T-lymphocyte antigen 4 genes as candidates for primary determinants of T2DM risk (18). Later, a landmark GWAS was conducted in seven common diseases, including T1DM and T2DM, among a large sample from the Wellcome Trust Case Control Consortium. The findings represented a thorough validation of the GWAS approach (19). Meanwhile, another GWAS in T2DM, also based on the Wellcome Trust Case Control Consortium, emphasized the contribution of multiple variants with modest effects on pathways influencing pancreatic beta cells and the etiology of T2DM (20). This study laid the foundation for comprehensive investigations in diabetic genetics. Other landmarks included DNA methylation in T1DM and its vascular complications, verification of the necessity for the comprehensive enumeration of sequence variations for the identification of functional alleles in multi-ancestry groups, and, last but not least, the 1000 Genomes Project. The genomes of 2,504 individuals from 26 populations were reconstructed to characterize a broad spectrum of genetic variations, displaying the distributions of genetic variations across the global sample (17, 21, 22). The findings indicated close global cooperation and revealed the epigenetic trends in diabetes in recent years.

In the author co-citation network, the first peak of citation appeared during 2007–2009. D. Altshuler, from the Broad Institute of Harvard and Massachusetts Institute of Technology (MIT), found that the human genome could be parsed into haplotype blocks, thereby laying the foundation for the construction of a haplotype map of the human genome in the early times (23). The researcher also did some pioneering work on the genetic mapping of complex traits in humans, which laid the foundation for GWAS in common diseases (24). Later on, from 2011 to 2012, D. Altshuler participated in the 1000 Genomes Project and provided insights into the characterization of the variations in human genome sequences (25). Furthermore, large-scale association analyses on lipid, blood pressure, and fasting glucose shed light on the pathophysiology of cardiovascular disease and T2DM (26–28). E. Zeggini, affiliated with the Wellcome Trust Centre for Human Genetics from the University of Oxford, reported a series of findings on GWAS. The researcher identified the susceptibility loci for T2DM (29, 30), including loci at or near the *FTO* gene (31), *MC4R* (32) influencing body mass, and *MTNR1B* associated with fasting glucose levels (33). This study was followed by a large-sample meta-analysis, including 21 GWAS in 46,186 non-diabetic participants, and verification of the susceptibility genes in an additional 76,558 participants (34). R. Saxena shared the contribution with E. Zeggini in genetic locus identification in T2DM (33, 34). Moreover, this author per se has identified the variants associated with lipid levels (35) and the risk of coronary artery disease (36). Thus, the citation counts for R. Saxena remained high during 2011–2014. In 2007, S. Purcell developed a famous toolset for GWAS and population-based linkage analyses, namely, PLINK (37). In recent years, he has endeavored to explain the genetic association in sleep disorders (38, 39). P.R. Burton, from the University of Leicester, elucidated the central concepts of modern genetic epidemiology and revealed its potential in devising appropriate preventive strategies for common diseases (40, 41). H. Li, from the Wellcome Trust Genome Campus affiliated with Cambridge, focused on mapping low-divergent sequences against a large reference genome and introducing the sequence alignment/map format as a universal tool for GWAS (42, 43).

In the hotspot analysis by timeline view of the keyword occurrence clustering, the largest cluster, epigenetics (cluster #0), contributed to the development of biological pathways influenced by metabolic risk factors (44). Preadipocytes from a person with T2DM demonstrated an intrinsic gene expression profile that persisted after several passes, highlighting that the early-life environment could affect adipose tissue phenotype (45). Other studies have demonstrated that alterations in DNA methylation are the consequence of adiposity (46, 47). Widespread changes in DNA methylation have been associated with body mass index, which predicts future T2DM (relative risk per 1 SD increase in the methylation risk score = 2.3, 95% CI = 2.07–2.56, *p* < 0.001) in an epigenome-wide association study including 10,261 samples (48), suggesting that DNA methylation is independent of the conventional risk factors toward predicting incident T2DM. Meanwhile, identification of methylation loci proved that genes are involved in lipid metabolism and inflammatory pathways, such as *ABCG1*, which is involved in insulin secretion and in the transport of cholesterol and phospholipids (49, 50). In addition, *TNFRSF4* and *MAP3K2* are involved in the activation of nuclear factor kappa B (NF-κB) (51).

For cluster #2 (genetic architecture), researchers have reported a greater number of loci associated with β-cell dysfunction (*KCNJ11, TCF7L2*, *KCNQ1*, *WFS1, HNF1B, SLC2A2, SLC30A8*, *ADRA2A, CAMK1D*, *CDKAL1*, *CDKN2A, CDKN2B*, *G6PC2, GLIS3, GCKR, GCK, IGF2BP2, NOTCH2, THADA*, *MTNR1B*, *PROX1*, and *GIPR*) and several loci associated with impaired insulin sensitivity and adiposity (*PPARG, IRS1, IGF1, FTO*, and *KLF14*) (34, 52, 53). Another GWAS using whole-genome sequencing in 2,657 European samples, exome sequencing in 12,940 individuals from five ancestries, and genotyping and imputation in a further 111,548 subjects provided proof that enumeration of genetic variations is necessary in order to identify functional alleles that provide important clues to the disease pathophysiology. However, it is difficult to explain the role of the less common variants (allele frequency <5%) in the predisposition to T2DM with the current approaches of GWAS (17).

## Limitations and Conclusion

There are some limitations to our study. Firstly, owing to the nature of the CiteSpace software, the present study could only index the references in the WoSCC database, which may not fully represent the available information in this field. Secondly, with the continuous update of the WoSCC database, non-English references were excluded and only published articles and reviews were included; therefore, a discrepancy may exist between our results and the overall publications. Finally, the distributions of cooperation among countries/regions and institutions were limited to GWAS in diabetes and, hence, do not apply to other common diseases.

In conclusion, the last two decades have witnessed a rising trend in the annual number of publications and citations on GWAS in diabetes. The current work has provided the history and trends of GWAS in diabetes and may enable the development of new strategies for the prediction and prevention of diabetes and other adverse clinical consequences. Further studies are warranted in the field of technical progress in GWAS to obtain detailed explanations concerning phenotypes that facilitate risk stratification and personalized medicine so as to tackle the current global epidemic of diabetes and its associated cardiovascular disturbances.

## Data Availability Statement

The original contributions presented in the study are included in the article/**Supplementary Material**. Further inquiries can be directed to the corresponding authors.

## Author Contributions

YL and YM conceived the study. YW and SQ retrieved the database. YL wrote the manuscript. WG revised the manuscript. All authors contributed to the article and approved the submitted version.

## Funding

The present work was supported by the Beijing Municipal Science & Technology Commission (project no. D141107005314004) and the Biotechnology Development Center of China (2016YFC1305200).

## Conflict of Interest

The authors declare that the research was conducted in the absence of any commercial or financial relationships that could be construed as a potential conflict of interest.

## Publisher’s Note

All claims expressed in this article are solely those of the authors and do not necessarily represent those of their affiliated organizations, or those of the publisher, the editors and the reviewers. Any product that may be evaluated in this article, or claim that may be made by its manufacturer, is not guaranteed or endorsed by the publisher.

## Abbreviations

GWAS, genome-wide association study; T1DM, type 1 diabetes mellitus; T2DM, type 2 diabetes mellitus; WoSCC, Web of Science Core Collection; LLR, log-likelihood rate; LRF, link-retaining factor; LBY, look-back years.

## References

[1] International Diabetes Federation. IDF Diabetes Atlas. 9th. Brussels: Belgium International Diabetes Federation (2019).

[2] Meigs JB Cupples LA Wilson PW. Parental Transmission of Type 2 Diabetes: The Framingham Offspring Study. Diabetes (2000) 12:2201–7. doi: 10.2337/diabetes.49.12.2201 11118026

[3] Mahajan A Taliun D Thurner M Robertson NR Torres JM Rayner NW. Fine-Mapping Type 2 Diabetes Loci to Single-Variant Resolution Using High-Density Imputation and Islet-Specific Epigenome Maps. Nat Genet (2018) 11:1505–13. doi: 10.1038/s41588-018-0241-6 PMC6287706 30297969

[4] Spracklen CN Horikoshi M Kim YJ Lin K Bragg F Moon S. Identification of Type 2 Diabetes Loci in 433,540 East Asian Individuals. Nature (2020) 7811:240–5. doi: 10.1038/s41586-020-2263-3 PMC7292783 32499647

[5] Vujkovic M Keaton JM Lynch JA Miller DR Zhou J Tcheandjieu C. Discovery of 318 New Risk Loci for Type 2 Diabetes and Related Vascular Outcomes Among 1.4 Million Participants in a Multi-Ancestry Meta-Analysis. Nat Genet (2020) 7:680–91. doi: 10.1038/s41588-020-0637-y PMC7343592 32541925

[6] McCarthy MI. Genomics, Type 2 Diabetes, and Obesity. N Engl J Med (2010) 24:2339–50. doi: 10.1056/NEJMra0906948 21142536

[7] Slatkin M. Linkage Disequilibrium–Understanding the Evolutionary Past and Mapping the Medical Future. Nat Rev Genet (2008) 6:477–85. doi: 10.1038/nrg2361 PMC5124487 18427557

[8] Chen C. Science Mapping: A Systematic Review of the Literature. J Data Inf Sci (2017) 2:1–40. doi: 10.1515/jdis-2017-0006

[9] Chen C CiteSpace II. Detecting and Visualizing Emerging Trends and Transient Patterns in Scientific Literature. J Am Soc Inf Sci Technol (2006) 3:359–77. doi: 10.1002/asi.20317

[10] Landis JR Koch GG. The Measurement of Observer Agreement for Categorical Data. Biometrics (1977) 1:159–74. doi: 10.2307/2529310 843571

[11] Freeman LC. Centrality in Social Networks Conceptual Clarification. Soc Netw (1979) 3:215–39. doi: 10.1016/0378-8733(78)90021-7

[12] Rousseeuw PJ. Silhouettes: A Graphical Aid to the Interpretation and Validation of Cluster Analysis. Comput Appl Math (1987) 20:53–65. doi: 10.1016/0377-0427(87)90125-7

[13] van Eck NJ Waltman L. Citation-Based Clustering of Publications Using CitNetExplorer and VOSviewer. Scientometrics (2017) 2:1053–70. doi: 10.1007/s11192-017-2300-7 PMC5400793 28490825

[14] Aryadoust V Zakaria A Lim MH Chen C. Corrigendum: An Extensive Knowledge Mapping Review of Measurement and Validity in Language Assessment and SLA Research. Front Psychol (2020) 11:643027. doi: 10.3389/fpsyg.2020.643027 33628195 PMC7898351

[15] Chen C. Searching for Intellectual Turning Points: Progressive Knowledge Domain Visualization. Proc Natl Acad Sci USA Suppl (2004) 1:5303–10. doi: 10.1073/pnas.0307513100 PMC387312 14724295

[16] Diabetes Genetics Initiative of Broad Institute of Harvard and MIT, Lund University, and Novartis Institutes of BioMedical Research Saxena R Voight BF Lyssenko V Burtt NP de Bakker PI Chen H. Genome-Wide Association Analysis Identifies Loci for Type 2 Diabetes and Triglyceride Levels. Science (2007) 5829:1331–6. doi: 10.1126/science.1142358 17463246

[17] Fuchsberger C Flannick J Teslovich TM Mahajan A Agarwala V Gaulton KJ. The Genetic Architecture of Type 2 Diabetes. Nature (2016) 7614:41–7. doi: 10.1038/nature18642 PMC5034897 27398621

[18] Ueda HH Joanna MM Esposito L Heward J Snook H Chamberlain G. Association of the T-Cell Regulatory Gene CTLA4 With Susceptibility to Autoimmune Disease. Nature (2003) 423:506–11. doi: 10.1038/nature01621 12724780

[19] Wellcome Trust Case Control Consortium. Genome-Wide Association Study of 14,000 Cases of Seven Common Diseases and 3,000 Shared Controls. Nature (2007) 7145:661–78. doi: 10.1038/nature05911 PMC2719288 17554300

[20] Zeggini E Weedon MN Lindgren CM Frayling TM Elliott KS Lango H. Replication of Genome-Wide Association Signals in UK Samples Reveals Risk Loci for Type 2 Diabetes. Science (2007) 5829:1336–41. doi: 10.1126/science.1142364 PMC3772310 17463249

[21] Agardh E Lundstig A Perfilyev A Volkov P Freiburghsaus T Lindholm E. Genome-Wide Analysis of DNA Methylation in Subjects With Type 1 Diabetes Identifies Epigenetic Modifications Associated With Proliferative Diabetic Retinopathy. BMC Med (2015) 13:182. doi: 10.1186/s12916-015-0421-5 26248552 PMC4527111

[22] 1000 Genomes Project Consortium Auton A Brooks LD Durbin RM Garrison EP Kang HM Korbel JO. A Global Reference for Human Genetic Variation. Nature (2015) 7571:68–74. doi: 10.1038/nature15393 PMC4750478 26432245

[23] Gabriel SB Schaffner SF Nguyen H Moore JM Roy J Blumenstiel B. The Structure of Haplotype Blocks in the Human Genome. Science (2002) 5576:2225–9. doi: 10.1126/science.1069424 12029063

[24] Altshuler D Daly MJ Lander ES. Genetic Mapping in Human Disease. Science (2008) 5903:881–8. doi: 10.1126/science.1156409 PMC2694957 18988837

[25] 1000 Genomes Project Consortium Abecasis GR Altshuler D Auton A Brooks LD Durbin RM Gibbs RA. A Map of Human Genome Variation From Population-Scale Sequencing. Nature (2010) 7319:1061–73. doi: 10.1038/nature09534 PMC3042601 20981092

[26] Teslovich TM Musunuru K Smith AV Edmondson AC Stylianou IM Koseki M. Biological, Clinical and Population Relevance of 95 Loci for Blood Lipids. Nature (2011) 7367:103–9. doi: 10.1038/nature10405 PMC3039276 20686565

[27] International Consortium for Blood Pressure Genome-Wide Association Studies Ehret GB Munroe PB Rice KM Bochud M Johnson AD Chasman DI. Genetic Variants in Novel Pathways Influence Blood Pressure and Cardiovascular Disease Risk. Nature (2011) 7367:103–9. doi: 10.1038/nature10405 PMC3340926 21909115

[28] Morris AP Voight BF Teslovich TM Ferreira T Segrè AV Steinthorsdottir V. Large-Scale Association Analysis Provides Insights Into the Genetic Architecture and Pathophysiology of Type 2 Diabetes. Nat Genet (2012) 9:981–90. doi: 10.1038/ng.2383 PMC3442244 22885922

[29] McCarthy MI Zeggini E. Genetics of Type 2 Diabetes. Curr Diabetes Rep (2006) 2:147–54. doi: 10.1007/s11892-006-0026-7 16542626

[30] McCarthy MI Zeggini E. Genome-Wide Association Scans for Type 2 Diabetes: New Insights Into Biology and Therapy. Trends Pharmacol Sci (2007) 12:598–601. doi: 10.1016/j.tips.2007.10.008 17997168

[31] Frayling TM Timpson NJ Weedon MN Zeggini E Freathy RM Lindgren CM. A Common Variant in the FTO Gene Is Associated With Body Mass Index and Predisposes to Childhood and Adult Obesity. Science (2007) 5826:889–94. doi: 10.1126/science.1141634 PMC2646098 17434869

[32] Loos RJ Lindgren CM Li S Wheeler E Zhao JH Prokopenko I. Common Variants Near MC4R Are Associated With Fat Mass, Weight and Risk of Obesity. Nat Genet (2008) 6:768–75. doi: 10.1038/ng.140 PMC2669167 18454148

[33] Prokopenko I Langenberg C Florez JC Saxena R Soranzo N Thorleifsson G. Variants in MTNR1B Influence Fasting Glucose Levels. Nat Genet (2009) 1:77–81. doi: 10.1038/ng.290 PMC2682768 19060907

[34] Dupuis J Langenberg C Prokopenko I Saxena R Soranzo N Jackson AU. New Genetic Loci Implicated in Fasting Glucose Homeostasis and Their Impact on Type 2 Diabetes Risk. Nat Genet (2010) 2:105–16. doi: 10.1038/ng.520 PMC3018764 20081858

[35] Willer CJ Schmidt EM Sengupta S Peloso GM Gustafsson S Kanoni S. Discovery and Refinement of Loci Associated With Lipid Levels. Nat Genet (2013) 11:1274–83. doi: 10.1038/ng.2797 PMC3838666 24097068

[36] Do R Willer CJ Schmidt EM Sengupta S Gao C Peloso GM. Common Variants Associated With Plasma Triglycerides and Risk for Coronary Artery Disease. Nat Genet (2013) 11:1345–52. doi: 10.1038/ng.2795 PMC3904346 24097064

[37] Purcell S Neale B Todd-Brown K Thomas L Ferreira MA Bender D. PLINK: A Tool Set for Whole-Genome Association and Population-Based Linkage Analyses. Am J Hum Genet (2007) 3:559–75. doi: 10.1086/519795 PMC1950838 17701901

[38] Lane JM Jones SE Dashti HS Wood AR Aragam KG van Hees VT. Biological and Clinical Insights From Genetics of Insomnia Symptoms. Nat Genet (2019) 3:387–93. doi: 10.1038/s41588-019-0361-7 PMC6415688 30804566

[39] Lane JM Liang J Vlasac I Anderson SG Bechtold DA Bowden J. Genome-Wide Association Analyses of Sleep Disturbance Traits Identify New Loci and Highlight Shared Genetics With Neuropsychiatric and Metabolic Traits. Nat Genet (2017) 2:274–81. doi: 10.1038/ng.3749 PMC5491693 27992416

[40] Davey Smith G Ebrahim S Lewis S Hansell AL Palmer LJ Burton PR. Genetic Epidemiology and Public Health: Hope, Hype, and Future Prospects. Lancet (2005) 9495:1484–98. doi: 10.1016/s0140-6736(05)67601-5 16243094

[41] Burton PR Tobin MD Hopper JL. Key Concepts in Genetic Epidemiology. Lancet (2005) 9489:941–51. doi: 10.1016/s0140-6736(05)67322-9 16154023

[42] Li H Durbin R. Fast and Accurate Short Read Alignment With Burrows-Wheeler Transform. Bioinformatics (2009) 14:1754–60. doi: 10.1093/bioinformatics/btp324 PMC2705234 19451168

[43] Li H Handsaker B Wysoker A Fennell T Ruan J Homer N. The Sequence Alignment/Map Format and SAMtools. Bioinformatics (2009) 16:2078–9. doi: 10.1093/bioinformatics/btp352 PMC2723002 19505943

[44] Portela A Esteller M. Epigenetic Modifications and Human Disease. Nat Biotechnol (2010) 10:1057–68. doi: 10.1038/nbt.1685 20944598

[45] van Tienen FH van der Kallen CJ Lindsey PJ Wanders RJ van Greevenbroek MM Smeets HJ. Preadipocytes of Type 2 Diabetes Subjects Display an Intrinsic Gene Expression Profile of Decreased Differentiation Capacity. Int J Obes (Lond) (2011) 9:1154–64. doi: 10.1038/ijo.2010.275 21326205

[46] Xu X Su S Barnes VA De Miguel C Pollock J Ownby D. A Genome-Wide Methylation Study on Obesity: Differential Variability and Differential Methylation. Epigenetics (2013) 5:522–33. doi: 10.4161/epi.24506 PMC3741222 23644594

[47] Feinberg AP Irizarry RA Fradin D Aryee MJ Murakami P Aspelund T. Personalized Epigenomic Signatures That Are Stable Over Time and Covary With Body Mass Index. Sci Transl Med (2010) 49:49ra67. doi: 10.1126/scitranslmed.3001262 PMC3137242 20844285

[48] Wahl S Drong A Lehne B Loh M Scott WR Kunze S. Epigenome-Wide Association Study of Body Mass Index, and the Adverse Outcomes of Adiposity. Nature (2017) 7635:81–6. doi: 10.1038/nature20784 PMC5570525 28002404

[49] Chambers JC Loh M Lehne B Drong A Kriebel J Motta V. Epigenome-Wide Association of DNA Methylation Markers in Peripheral Blood From Indian Asians and Europeans With Incident Type 2 Diabetes: A Nested Case-Control Study. Lancet Diabetes Endocrinol (2015) 7:526–34. doi: 10.1016/s2213-8587(15)00127-8 PMC4724884 26095709

[50] Hidalgo B Irvin MR Sha J Zhi D Aslibekyan S Absher D. Epigenome-Wide Association Study of Fasting Measures of Glucose, Insulin, and HOMA-IR in the Genetics of Lipid Lowering Drugs and Diet Network Study. Diabetes (2014) 2:801–7. doi: 10.2337/db13-1100 PMC3968438 24170695

[51] Karin M Ben-Neriah Y. Phosphorylation Meets Ubiquitination: The Control of NF-[Kappa]B Activity. Annu Rev Immunol (2000) 18:621–63. doi: 10.1146/annurev.immunol.18.1.621 10837071

[52] Voight BF Scott LJ Steinthorsdottir V Morris AP Dina C Welch RP. Twelve Type 2 Diabetes Susceptibility Loci Identified Through Large-Scale Association Analysis. Nat Genet (2010) 7:579–89. doi: 10.1038/ng.609 PMC3080658 20581827

[53] Saxena R Hivert MF Langenberg C Tanaka T Pankow JS Vollenweider P. Genetic Variation in GIPR Influences the Glucose and Insulin Responses to an Oral Glucose Challenge. Nat Genet (2010) 2:142–8. doi: 10.1038/ng.521 PMC2922003 20081857

