# Supplementary material for: Insights Into Genome-Wide Association Study for Diabetes: A Bibliometric and Visual Analysis From 2001 to 2021
Source: Front Endocrinol (Lausanne). 2022 Mar 11;13:817620. doi: 10.3389/fendo.2022.817620 (PMC8963272; doi:10.3389/fendo.2022.817620)
Supplement: Supplementary file 1 [file DataSheet_1.docx]

**Betweeness centrality:**

Betweeness centrality is an index to measure the importance of nodes in a network. This metric is used in the CiteSpace software to discover and measure the importance of documents, with the purple circle emphasizing documents (or authors, journals, and institutions, etc.) such that the centrality of the node with a purple ring shall be ≥0.1). Literature with a high betweeness centrality often serves as the key hub connecting two different areas, also known as the turning points in CiteSpace.

The formula used to calculate the betweeness centrality was proposed by Freeman in 1977[1], as follows:


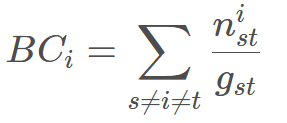


Where, gst is the number of shortest paths from node s to node t, nist is the number of shortest paths passing through node i in the gst shortest paths from node s to node t. From the perspective of information transmission, the higher the betweeness centrality, greater is the importance of node and greater is the impact on network transmission after removing this particular node.

**Silhouette value:**

The silhouette is a measure of how closely it is matched to data within its cluster and how loosely it is matched to the data of the neighboring cluster, that is the homogeneity in a word [2]. The greater the value, higher is the similarity between the internal members of the class and better is the consistency. The silhouette >0.5 indicates that clustering is reasonable and that value >0.7 represents a high-quality network. The silhouette value of each sample point is calculated, as follows:


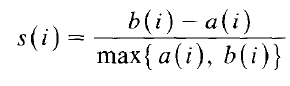


Where, “a” is the average distance between point i and other points in the cluster; “b” is the average distance between point i and other points closet to point i in the cluster. The mean silhouette score is the average of the silhouette values of each sample point.

**Modularity Q:**

Modularity Q is the metric used to evaluate modularization. Low modularity Q reveals that the clustering content is loose and incomplete. The value range of modularity Q is [0,1], while modularity Q >0.3 indicates that the network community structure is significant and that the network is convincing. Modularity Q value is calculated, as follows [3]:


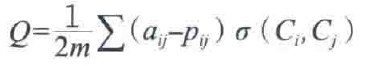


Where, A = aij is the adjacency matrix of the actual network; pij is the expected value of the number of connecting lines between node i and node j in the zero model; C i and C j represents the communities to which node i and node j belong in a network. If i and j belong to the same community, σ = 1; otherwise σ = 0.

**Harmonic mean:** The mean of n numbers expressed as the reciprocal of the arithmetic mean of the reciprocals of all numbers.

**Pruning pathfinder:** The process to systematically remove excessive links is called network pruning. The CiteSpace provides two ways for this purpose: Pathfinder and Minimum Spanning Tree. A comparison of the pros and cons of the two methods is detailed in a 2003 publication [4]. In a nutshell, Pathfinder is a theoretically better option, albeit slightly expensive.

**Algorithm log-likelihood rate (LLR):**

The likelihood ratio (LR) is an index reflecting authenticity that belongs to a composite index simultaneously reflecting sensitivity and specificity. Ted Dunning [5] introduced the basic use of log–likelihood ratio (LLR) in text calculation. LLR can be appropriately used in industrial systems as it is simple, can be effectively implemented, and scalable on large-scale data. Therefore, LLR based similarity measurement is widely employed in several applications, such as the recommendation system. In CiteSpace, after adjusting for the clustering of co-citation networks to obtain a satisfactory map, different methods can be used to name the clusters. Past studies have reported that LLR usually provides the best outcomes in terms of uniqueness and coverage in clustering analyses [6].

**Top N:** In the selection criteria, Top 100 per slice indicates that the top 100 data of each time-slice can be extracted to generate the final network (the specific meaning of Top100 varies with the node types selected). For example, if the author is selected for cooperative analysis, the author of Top100 in the number of documents published in this time period is extracted. For CO citation analysis, the data of Top100 in each time slice is extracted) [6].

**Link-retaining factor (LRF):** This parameter adjusts the choice of link. It retains the strongest link K-times the network size and eliminates the remaining links [3].

**Look back years (LBY):** The time span of the adjustment link is no more than N years [3].

**e:** Top N= {v|f (V) > = min (f (top (n), e)}, is the setting of the lowest frequency of the node. [3]

1. L. C. Freeman. Centrality in Social Networks Conceptual Clarification. *Social Networks*,1979, 3:215-239. https://doi.org/10.1016/0378-8733(78)90021-7

2. Peter J. Rousseuw. “Silhouettes: a Graphical Aid to the Interpretation and Validation of Cluster Analysis”. *Computational and Applied Mathematics*, 1987, 20: 53–65. <https://doi.org/10.1016/0377-0427(87)90125-7>

3. Jie Li, and Chaomei Chen. CiteSpace: Text Mining and Visualization in Scientific Literature (Second Edition). Capital University of Economics and Trade Press, 2016. isbn:978-7-5638-2683-4.

4. Chaomei Chen ; Morris. Visualizing evolving networks: minimum spanning trees versus pathfinder networks. *IEEE Symposium on Information Visualization* 2003, 2003:67-74.

5. Dunning, T. Accurate methods for the statistics of surprise and coincidence. *Comput. Linguist*,1993, 19, 1:61-74.

6. Chaomei Chen. The CiteSpace Manual, 2014. [http://cluster.ischool.drexel.edu/~cchen/CiteSpace/CiteSpaceManual.pdf](http://cluster.ischool.drexel.edu/~cchen/citespace/CiteSpaceManual.pdf)
