# Supplementary material for: Insights Into Genome-Wide Association Study for Diabetes: A Bibliometric and Visual Analysis From 2001 to 2021
Source: Front Endocrinol (Lausanne). 2022 Mar 11;13:817620. doi: 10.3389/fendo.2022.817620 (PMC8963272; doi:10.3389/fendo.2022.817620)
Supplement: Supplementary file 2 [file Table_1.docx]

**Table S1 Top 10 clusters in keywords co-occurrence network**

| **Cluster ID** | **Size** | **Silhouette** | **mean(Year)** | **Label (LLR)** |
| --- | --- | --- | --- | --- |
| 0 | 53 | 0.803 | 2012 | epigenetics (37.34, 1.0E-4); dna methylation (24.27, 1.0E-4); metabolic memory (21.43, 1.0E-4); diabetic complications (15.19, 1.0E-4); histone modifications (10.68, 0.005); adipocytes (10.68, 0.005); apolipoprotein a ii (9.99, 0.005); epigenomics (8.61, 0.005); diabetes (6.4, 0.05); atherosclerosis (6.35, 0.05); qtl (6.35, 0.05); genome (5.27, 0.05); adipose tissue (5.27, 0.05); calpain10 (4.99, 0.05); clinical pathogen (4.99, 0.05); transforming growth factor beta-1 (4.99, 0.05); janibacter melonis (4.99, 0.05); diabetic kidney disease (4.99, 0.05); atherosclerosis susceptibility (4.99, 0.05); early onset obesity (4.99, 0.05); ppar (4.99, 0.05); gene map (4.99, 0.05); maltreatment (4.99, 0.05); weight (4.99, 0.05); diabetes complications (4.99, 0.05); iowa adoptee sample (4.99, 0.05); body fat (4.99, 0.05); ten-eleven translocation-2 protein (4.99, 0.05); diabetic complication (4.99, 0.05); txnip (4.99, 0.05); experience (4.99, 0.05); depression (4.99, 0.05); glucocorticoid receptor (4.99, 0.05); profile (4.99, 0.05); epigenetic therapies (4.99, 0.05); search (4.99, 0.05); tak1 (4.99, 0.05); small rna sequencing (4.99, 0.05); prospective (4.99, 0.05); omental (4.99, 0.05); very low density (4.99, 0.05); breast cancer (4.99, 0.05); mesangial cells (4.99, 0.05); expression data (4.99, 0.05); biochemistry (4.99, 0.05); genome sequencing (4.99, 0.05); factor kappa b (4.99, 0.05); sexual abuse (4.99, 0.05); vascular dysfunction (4.99, 0.05); diabetic cardiomyopathy (4.99, 0.05); vascular complications (4.99, 0.05); virulence (4.99, 0.05); renal hypoxia (4.99, 0.05); tokushima fatty rat (4.99, 0.05); proliferative retinopathy (4.99, 0.05); non-coding rna (4.99, 0.05); plasma lipoprotein (4.99, 0.05); oletf rat (4.99, 0.05); 3t3-l1 (4.99, 0.05); reveal (4.99, 0.05); visfatin (4.99, 0.05); glucose homeostasis (4.99, 0.05); endothelial cell (4.99, 0.05); jazf1 (4.99, 0.05); food intake (4.99, 0.05); association (4.82, 0.05); high glucose (4.79, 0.05); hyperglycemia (4.79, 0.05); methylation (4.57, 0.05); expression (3.77, 0.1); gene (3.42, 0.1); microrna (3.04, 0.1); susceptibility (2.73, 0.1); diabetes mellitus (2.56, 0.5); diabetic nephropathy (2.47, 0.5); twins (2.39, 0.5); next generation sequencer (2.39, 0.5); acetylation (2.39, 0.5); epigenetic marks (2.39, 0.5); chromatin state (2.39, 0.5); histone acetylation (2.39, 0.5); muscle (2.39, 0.5); histone (2.39, 0.5); next-generation sequencing (2.39, 0.5); high throughput sequencing (2.39, 0.5); vascular cells (2.39, 0.5); complex (2.39, 0.5); personalized management (2.39, 0.5); go analysis (2.39, 0.5); histone methylation (2.39, 0.5); chip-seq (2.39, 0.5); faire-seq (2.39, 0.5); podocyte (2.39, 0.5); chronic diabetic complications (2.39, 0.5); ingenuity pathway analysis (2.39, 0.5); posttraumatic stress disorder (2.39, 0.5); non coding rnas (2.39, 0.5); high-fat diet (2.39, 0.5); histone deacetylase (2.39, 0.5); cholesterol (2.39, 0.5) |
| 1 | 44 | 0.856 | 2006 | mellitus (16.22, 1.0E-4); linkage (11.31, 0.001); candidate genes (9.77, 0.005); body mass index (8.55, 0.005); disease (8.55, 0.005); animal model (8.43, 0.005); bioinformatics (8.42, 0.005); diabetes (7.79, 0.01); gene (7.04, 0.01); epigenetics (5.68, 0.05); genome wide association (4.9, 0.05); human disease (4.87, 0.05); complex trait (4.87, 0.05); genetic epidemiology (4.87, 0.05); functional genomics (4.21, 0.05); alstrom syndrome (4.21, 0.05); functional annotation (4.21, 0.05); fatty liver (4.21, 0.05); familial genetic evaluation (4.21, 0.05); indian scenario (4.21, 0.05); xp11.3-xp11.23 (4.21, 0.05); cone-rod dystrophy (4.21, 0.05); in vitro (4.21, 0.05); hepatic nuclear factor 1 beta (hnf1 beta) (4.21, 0.05); hyperphenylalaninemia (4.21, 0.05); genome-wide association scan (gwas) (4.21, 0.05); joubert syndrome (4.21, 0.05); congenital nephrotic syndrome (4.21, 0.05); pig (4.21, 0.05); polycystic kidney disease (4.21, 0.05); proliferator activated receptor gamma 2 (4.21, 0.05); human insulin gene (4.21, 0.05); diabetic foot infection (4.21, 0.05); melanocortin (4.21, 0.05); novel genes (4.21, 0.05); neurogenetic disorders (4.21, 0.05); pseudogenes (4.21, 0.05); maturity-onset diabetes of the young type 5 (mody5) (4.21, 0.05); penis (4.21, 0.05); polymorphism single nucleotide (4.21, 0.05); genetic variants (4.21, 0.05); h 2 (4.21, 0.05); insulin deficiency (4.21, 0.05); hepatocyte nuclear factor 4 alpha (4.21, 0.05); genome screening (4.21, 0.05); fawn hooded rat (4.21, 0.05); peroxisome proliferatoractivated (4.21, 0.05); inherited kidney disease (4.21, 0.05); association studies (4.21, 0.05); tallyho/jng mice (4.21, 0.05); antioxidant vitamin (4.21, 0.05); epigenetic epidemiology (4.21, 0.05); alms1 gene (4.21, 0.05); genetic dissection (4.21, 0.05); heterogeneity (4.21, 0.05); meth-qtls (4.21, 0.05); in silico sequence analysis (4.21, 0.05); worldwide (4.21, 0.05); ethnic variability (4.21, 0.05); human adipose tissue (4.21, 0.05); genome wide search (4.21, 0.05); aging (4.21, 0.05); sensitive k channel (4.21, 0.05); nephrin localize (4.21, 0.05); regulation of transcription (4.21, 0.05); hepatic steatosis (4.21, 0.05); us adult (4.21, 0.05); body specific methylation (4.21, 0.05); heritable renal disease (4.21, 0.05); microarray analysis (4.21, 0.05); segregation analysis (4.21, 0.05); hyperglycaemia (4.21, 0.05); leptin (4.21, 0.05); chromosome 19q13 (4.21, 0.05); integrated gene map (4.21, 0.05); translational medicine (4.21, 0.05); dehydratase (4.21, 0.05); c-peptide (4.21, 0.05); unigene clusters (4.21, 0.05); dna methylation change (4.21, 0.05); focal segmental glomerulosclerosis (4.21, 0.05); peripheral blood (4.21, 0.05); hispanics (4.21, 0.05); genomewide association study (4.21, 0.05); prospective cohort (4.21, 0.05); loki (4.21, 0.05); genetics of diabetes (4.21, 0.05); enhancer (4.21, 0.05); erectile dysfunction (4.21, 0.05); diabetes mellitus type i (4.21, 0.05); iodine (4.21, 0.05); genetic engineering (4.21, 0.05); maturity-onset diabetes of the young (mody) (4.21, 0.05); mitochondrial ribosomal proteins (4.21, 0.05); cell type (4.21, 0.05); diabetes mellitus iddm (4.21, 0.05); quantitative trait (4.21, 0.05); dcoh (4.21, 0.05); hl a antigen (4.21, 0.05); whole-genome re-sequencing (4.21, 0.05) |
| 2 | 38 | 0.749 | 2013 | genetic architecture (13.86, 0.001); rare variant (13.86, 0.001); genome wide association (13.49, 0.001); heritability (9.55, 0.005); open chromatin (9.23, 0.005); risk prediction (9.23, 0.005); common disease (9.23, 0.005); regression (9.23, 0.005); rare variants (9.23, 0.005); heart disease (9.23, 0.005); wide association (6.16, 0.05); annotation (5.62, 0.05); framework (5.62, 0.05); complex traits (5.62, 0.05); model (5.62, 0.05); discovery (5.62, 0.05); childhood (5.62, 0.05); mutation (5.52, 0.05); type 2 (4.61, 0.05); variable selection (4.61, 0.05); sequence variation (4.61, 0.05); functional data analysis (4.61, 0.05); blood (4.61, 0.05); kernel test (4.61, 0.05); albinism (4.61, 0.05); mirna (4.61, 0.05); alignment (4.61, 0.05); array cgh (4.61, 0.05); protein complexe (4.61, 0.05); rice (4.61, 0.05); gwa (4.61, 0.05); full genome (4.61, 0.05); cox (4.61, 0.05); multiethnic cohort (4.61, 0.05); bilateral facial palsy (4.61, 0.05); gene gene interaction (4.61, 0.05); dipeptidyl peptidase 4 (4.61, 0.05); binding site (4.61, 0.05); disease association study (4.61, 0.05); ischemic stroke (4.61, 0.05); personal genomics (4.61, 0.05); hospitalization data (4.61, 0.05); pseudomonas (4.61, 0.05); ld block (4.61, 0.05); bayesian methods (4.61, 0.05); cystic fibrosis (4.61, 0.05); physicochemical property (4.61, 0.05); family history (4.61, 0.05); tissue (4.61, 0.05); low frequency (4.61, 0.05); axi (4.61, 0.05); deletion (4.61, 0.05); ethanol production (4.61, 0.05); synthase i (4.61, 0.05); genotype 4 (4.61, 0.05); architecture (4.61, 0.05); hemizygosity (4.61, 0.05); disorder (4.61, 0.05); diabetic foot ulcer (4.61, 0.05); epistasis (4.61, 0.05); ciprofloxacin (4.61, 0.05); population differentiation (4.61, 0.05); imputation (4.61, 0.05); sequencing data (4.61, 0.05); amylose extender mutation (4.61, 0.05); burden test (4.61, 0.05); cardiovascular risk (4.61, 0.05); pax6 (4.61, 0.05); regulatory element (4.61, 0.05); population scale (4.61, 0.05); carbapenem (4.61, 0.05); management (4.61, 0.05); validation (4.61, 0.05); snp heritability (4.61, 0.05); african genetic diversity (4.61, 0.05); electronic health record (4.61, 0.05); genomic medicine (4.61, 0.05); whole blood (4.61, 0.05); cardiovascular risk factors (4.61, 0.05); molecular basis (4.61, 0.05); branching enzyme iib (4.61, 0.05); extrahepatic manifestation (4.61, 0.05); paraoxonase activity (4.61, 0.05); family study (4.61, 0.05); hybrid design (4.61, 0.05); range chromosomal interaction (4.61, 0.05); identify patient (4.61, 0.05); metabolic disease (4.61, 0.05); polygenic risk (4.61, 0.05); alcohol (4.61, 0.05); outcm (4.61, 0.05); disease susceptibility (4.61, 0.05); association mapping (4.61, 0.05); administrative data (4.61, 0.05); metsim (4.61, 0.05); glucose tolerance (4.61, 0.05); susceptibility loci (4.61, 0.05); biofilm (4.61, 0.05); common variants (4.61, 0.05); mouse chromosome 2 (4.61, 0.05) |
| 3 | 37 | 0.926 | 2007 | type 2 diabetes (16.34, 1.0E-4); transcription factor foxo1 (11.4, 0.001); autosomal genomic scan (11.4, 0.001); bone mineral density (11.4, 0.001); insulin resistance (8.52, 0.005); region (7.7, 0.01); genome-wide association studies (6.1, 0.05); triacylglycerol synthesis (5.69, 0.05); familial resemblance (5.69, 0.05); fat mas (5.69, 0.05); lncrna (5.69, 0.05); hl a (5.69, 0.05); extreme phenotypes (5.69, 0.05); mass deposition (5.69, 0.05); gene-targeted mice (5.69, 0.05); sasp (5.69, 0.05); colorectal cancer (5.69, 0.05); acid response element (5.69, 0.05); dna microarray (5.69, 0.05); apc (5.69, 0.05); lipodystrophy (5.69, 0.05); age-related diseases (5.69, 0.05); metabolic complications (5.69, 0.05); arab population (5.69, 0.05); risk locus (5.69, 0.05); triglyceride (5.69, 0.05); nuclear factor 3 (5.69, 0.05); late gestation (5.69, 0.05); vitamin a (5.69, 0.05); essential hypertension (5.69, 0.05); african american pedigree (5.69, 0.05); genome-wide association study (5.69, 0.05); target gene discovery (5.69, 0.05); promoter (5.69, 0.05); hdac3 (5.69, 0.05); insulin-like growth factor i (5.69, 0.05); assessing genetic linkage (5.69, 0.05); nutrigenetics (5.69, 0.05); stk39 (5.69, 0.05); homogeneous (5.69, 0.05); global burden (5.69, 0.05); serum amyloid a (5.69, 0.05); longitudinal change (5.69, 0.05); tissue-specific expression (5.69, 0.05); adenoma (5.69, 0.05); inflammatory bowel disease (5.69, 0.05); centenarians (5.69, 0.05); bipolar affective disorder (5.69, 0.05); phosphoenolpyruvate carboxykinase gene (5.69, 0.05); agpat2 (5.69, 0.05); pharmacogenetics (5.69, 0.05); body composition (5.69, 0.05); major susceptibility locus (5.69, 0.05); consanguineous (5.69, 0.05); postmenopausal caucasian women (5.69, 0.05); resistance (5.69, 0.05); old order amish (5.69, 0.05); sensitivity (5.69, 0.05); adipocyte differentiation (5.69, 0.05); spak (5.69, 0.05); whole-genome scan (5.69, 0.05); alcohol consumption (5.69, 0.05); endeavour (5.69, 0.05); dependent diabetes mellitus (4.24, 0.05); heritability (3.62, 0.1); diabetes (3.59, 0.1); allele frequencies (3.04, 0.1); differentially methylated genes (3.04, 0.1); risk score (3.04, 0.1); pancreatic islets (3.04, 0.1); tcf7l2 (3.04, 0.1); pattern (3.04, 0.1); hla non-classical loci (3.04, 0.1); genetic basis (3.04, 0.1); systematic review (3.04, 0.1); transcription factors (3.04, 0.1); haplotype frequencies (3.04, 0.1); pima indian (3.04, 0.1); united states (3.04, 0.1); latin americans (3.04, 0.1); gene ontology enrichment analysis (3.04, 0.1); genetics (2.62, 0.5); epigenetics (2.62, 0.5); quantitative trait loci (2.34, 0.5); onset (2.12, 0.5); lipid metabolism (2.12, 0.5); genes (2.12, 0.5); human disease (2.12, 0.5); hispanic (2.12, 0.5); complex traits (2.12, 0.5); allele (2.12, 0.5); population genetics (2.12, 0.5); genotype (2.12, 0.5); risk (2.04, 0.5); gene expression (1.9, 0.5); family (1.55, 0.5); systemic lupus erythematosus (1.55, 0.5); blood pressure (1.55, 0.5); prediction (1.55, 0.5); single nucleotide polymorphism (1.53, 0.5) |
| 4 | 35 | 0.847 | 2010 | expression (11.07, 0.001); insulin secretion (9.54, 0.005); er stress (9.21, 0.005); mice (9.21, 0.005); b cell (9.21, 0.005); risk loci (6.14, 0.05); translation (5.6, 0.05); in vivo (5.6, 0.05); activation (5.5, 0.05); identification (5.5, 0.05); variant (4.85, 0.05); islets (4.6, 0.05); biosynthetic gene cluster (4.6, 0.05); lineage commitment (4.6, 0.05); amino acid transporter (4.6, 0.05); transcription factor nkx6.1 (4.6, 0.05); sterol-regulated (4.6, 0.05); mutagenesis (4.6, 0.05); tallyho (4.6, 0.05); sequence deletion (4.6, 0.05); interferon gamma (4.6, 0.05); cd4(+) t cell (4.6, 0.05); wolfram syndrome (4.6, 0.05); glucolipotoxicity (4.6, 0.05); rkip (4.6, 0.05); hepatic encephalopathy (4.6, 0.05); embryonic stem cell (4.6, 0.05); hypertrophic cardiomyopathy (4.6, 0.05); akt (4.6, 0.05); pten (4.6, 0.05); snps (4.6, 0.05); gene expression profiling (4.6, 0.05); t1d (4.6, 0.05); insulin synthesis (4.6, 0.05); neonatal beta cell growth (4.6, 0.05); saccharomyces cerevisiae (4.6, 0.05); pi3k (4.6, 0.05); pi3k/pten/akt/mtor pathway (4.6, 0.05); wfs1 gene (4.6, 0.05); autosomal recessive (4.6, 0.05); neurohypophyseal (4.6, 0.05); diphenyl ether (4.6, 0.05); p53 (4.6, 0.05); transcription factor gata 3 (4.6, 0.05); intragenic binding (4.6, 0.05); recessive syndrome (4.6, 0.05); glutamine (4.6, 0.05); tetracenomycin c (4.6, 0.05); apolipoprotein(a) (4.6, 0.05); marker (4.6, 0.05); acarviose metabolite production (4.6, 0.05); glucose infusion (4.6, 0.05); cd4 t cell (4.6, 0.05); insulin-secreting cells (4.6, 0.05); craniosynostosis (4.6, 0.05); genes immediate-early (4.6, 0.05); beta-cells (4.6, 0.05); oligonucleotide array sequence analysis (4.6, 0.05); endoplasmic reticulum (er) (4.6, 0.05); phlpp (4.6, 0.05); acarbose biosynthesis gene cluster (4.6, 0.05); fatty acids nonesterified (4.6, 0.05); interpreting transcriptional change (4.6, 0.05); 5`-hydroxy streptomycin (4.6, 0.05); bet (4.6, 0.05); gene silencing (4.6, 0.05); adipoq (4.6, 0.05); mitochondrial dysfunction (4.6, 0.05); pancreas (4.6, 0.05); proliferation (4.6, 0.05); adult rat (4.6, 0.05); cdk5 (4.6, 0.05); health (4.6, 0.05); raf-1 (4.6, 0.05); palmitates (4.6, 0.05); pbde (4.6, 0.05); hcfc1 (4.6, 0.05); array cgh analysis (4.6, 0.05); nf-kappa b (4.6, 0.05); cdkal1 (4.6, 0.05); histone h3 (4.6, 0.05); congenic mice (4.6, 0.05); proinsulin (4.6, 0.05); ifn gamma (4.6, 0.05); avp gene (4.6, 0.05); polychlorinated biphenyl (4.6, 0.05); streptomyces glaucescens (4.6, 0.05); persistent organic pollutant (4.6, 0.05); polarization (4.6, 0.05); cholesterol homeostasis (4.6, 0.05); bde 47 (4.6, 0.05); hek cells (4.6, 0.05); diabetes insipidus (4.6, 0.05); molecular mechanism (4.6, 0.05); laboratory mouse (4.6, 0.05); family (4.09, 0.05); nf kappa b (4.09, 0.05); autoimmune disease (4.09, 0.05); metabolic syndrome (3.63, 0.1); protein (3.11, 0.1) |
| 5 | 35 | 0.968 | 2005 | bipolar disorder (17.93, 1.0E-4); cognition (8.22, 0.005); diabetes mellitus (7.66, 0.01); arc protein (5.96, 0.05); dementia (5.96, 0.05); major depressive disorder (5.96, 0.05); molecular epidemiology (5.96, 0.05); renal failure (5.96, 0.05); allelic association (5.96, 0.05); parkinson disease (5.96, 0.05); genetic techniques (5.96, 0.05); peripheral blood mononuclear cells (5.96, 0.05); osteoporosis (5.96, 0.05); magnetic resonance imaging (5.96, 0.05); cohort study (5.96, 0.05); insulin receptor gene (5.96, 0.05); x chromosome inactivation (5.96, 0.05); personalized medicine (5.96, 0.05); necrosis factor alpha (5.96, 0.05); memory (5.96, 0.05); severity (5.96, 0.05); calcium (5.96, 0.05); pharmaco-epidemiology (5.96, 0.05); hashimotos thyroiditi (5.96, 0.05); macular degeneration (5.96, 0.05); t lymphocyte antigen 4 (5.96, 0.05); causality (5.96, 0.05); heart failure (5.96, 0.05); intracellular signal transduction (5.96, 0.05); diabetes genetics (5.96, 0.05); epidemlologic methods (5.96, 0.05); whole genome expression (5.96, 0.05); kraepelinian dichotomy (5.96, 0.05); dna extraction (5.96, 0.05); cohort studies (5.96, 0.05); plasminogen activator inhibitor 1 (5.96, 0.05); complex segregation analysis (5.96, 0.05); chromosome 20 (5.96, 0.05); transcript map (5.96, 0.05); stroke (5.96, 0.05); low birth weight (5.96, 0.05); oral disease (5.96, 0.05); brain-derived neurotrophic factor (5.96, 0.05); case-control studies (5.96, 0.05); rolipram (5.96, 0.05); oral microbiome (5.96, 0.05); cag repeat polymorphism (5.96, 0.05); gene frequency (5.96, 0.05); sirt1 deacetylase (5.96, 0.05); systemic disease (5.96, 0.05); serotonin (5.96, 0.05); alzheimers disease (5.96, 0.05); cell survival (5.96, 0.05); glaucoma (5.96, 0.05); physical map (5.96, 0.05); alzheimer disease (5.96, 0.05); thyroglobulin gene (5.96, 0.05); messenger ribonucleic acid (5.96, 0.05); nitric oxide (5.96, 0.05); mood disorder (5.96, 0.05); camp response element-binding protein (5.96, 0.05); congenital adrenal hyperplasia (5.96, 0.05); genomics software (5.96, 0.05); genome wide analysis (5.51, 0.05); single nucleotide polymorphism (4.91, 0.05); molecular genetics (4.71, 0.05); type 2 diabetes (3.35, 0.1); iddm8 (3.29, 0.1); allele frequencies (3.29, 0.1); triplet repeats (3.29, 0.1); degree relative (3.29, 0.1); caa repeats (3.29, 0.1); creb (3.29, 0.1); genetic association (3.29, 0.1); cag repeats (3.29, 0.1); japanese population (3.29, 0.1); hla non-classical loci (3.29, 0.1); myocardial infarction (3.29, 0.1); schizophrenia (3.29, 0.1); strain (3.29, 0.1); complex (3.29, 0.1); transmission disequilibrium test (3.29, 0.1); genetic heterogeneity (3.29, 0.1); end-stage renal disease (3.29, 0.1); follow up (3.29, 0.1); meta-analysis (3.29, 0.1); type i diabetes (3.29, 0.1); dna (3.29, 0.1); haplotype frequencies (3.29, 0.1); tata box-binding protein (3.29, 0.1); type 2 diabetes mellitus (3.29, 0.1); mitochondrial dna (3.29, 0.1); albuminuria (3.29, 0.1); tbp (3.29, 0.1); genotyping (2.35, 0.5); prostate (2.35, 0.5); transcription factor (2.35, 0.5); mitochondrial biogenesis (2.35, 0.5); population genetics (2.35, 0.5); genome-wide analysis (2.35, 0.5) |
| 6 | 33 | 0.93 | 2014 | association (18.8, 1.0E-4); type 1 diabetes (10.45, 0.005); duck (10.45, 0.005); domestication (10.45, 0.005); artificial selection (10.45, 0.005); hla (7.83, 0.01); database (6.78, 0.01); parabacteroides distasonis (5.22, 0.05); znt8 (5.22, 0.05); sleep (5.22, 0.05); behavior (5.22, 0.05); steatosis (5.22, 0.05); invasion (5.22, 0.05); population history (5.22, 0.05); screening (5.22, 0.05); acid (5.22, 0.05); skeleton (5.22, 0.05); complaint (5.22, 0.05); lysg-p-lyse (5.22, 0.05); carcinoma (5.22, 0.05); methionine metabolism (5.22, 0.05); programming evolution (5.22, 0.05); steatohepatiti (5.22, 0.05); molecular mimicry (5.22, 0.05); diversity (5.22, 0.05); southwest (5.22, 0.05); bacteroides uniformis (5.22, 0.05); genetic linkage (5.22, 0.05); precision medicine (5.22, 0.05); politics (5.22, 0.05); chromosome (5.22, 0.05); adaptive laboratory evolution (5.22, 0.05); motor restlessness (5.22, 0.05); autoimmune thyroiditis (5.22, 0.05); sequence analysis (5.22, 0.05); sex (5.22, 0.05); methylglyoxal (5.22, 0.05); 4-hydroxyisoleucine (5.22, 0.05); pair analysis (5.22, 0.05); middle age (5.22, 0.05); immune cell signatures (5.22, 0.05); triglycerides (5.22, 0.05); deep phenotyping (5.22, 0.05); bmd (5.22, 0.05); module map (5.22, 0.05); connective tissue growth factor (5.22, 0.05); phocaeicola dorei (5.22, 0.05); dispersal (5.22, 0.05); adaptation (5.22, 0.05); lys biosensor (5.22, 0.05); genome screen (5.22, 0.05); phage (5.22, 0.05); insulin signaling pathway (5.22, 0.05); fracture risk (5.22, 0.05); transcriptome analysis (5.22, 0.05); advanced imaging (5.22, 0.05); fibrosis (5.22, 0.05); signature (5.22, 0.05); gestational diabetes (5.22, 0.05); celiac disease (5.22, 0.05); corynebacterium glutamicum (5.22, 0.05); chronic hepatitis c (5.22, 0.05); 12/15 lipoxygenase (5.22, 0.05); modern human (5.22, 0.05); mitochondria (5.22, 0.05); susceptibility locus (5.21, 0.05); metabolism (4.17, 0.05); expression (3.35, 0.1); epigenetics (3.35, 0.1); obesity (3.35, 0.1); genome wide association (2.89, 0.1); metabolomics (2.6, 0.5); iddm4 (2.6, 0.5); affected sib pair (2.6, 0.5); gout (2.6, 0.5); progression (2.6, 0.5); insulin gene region (2.6, 0.5); national health insurance (2.6, 0.5); hashimotos thyroiditis (2.6, 0.5); genome-wide analysis study (2.6, 0.5); mexican american (2.6, 0.5); incidence (2.6, 0.5); outpatient records (2.6, 0.5); diet (2.6, 0.5); trait (2.6, 0.5); iddm2 locus (2.6, 0.5); fatty acids (2.6, 0.5); bb rat (2.6, 0.5); snp (2.6, 0.5); human thymus (2.6, 0.5); whole-genome dna methylation analysis (2.6, 0.5); early onset (2.6, 0.5); lasso (2.6, 0.5); genetic risk factor (2.6, 0.5); mitochondrial dna (2.6, 0.5); grass carp (2.6, 0.5); type 1 diabetes mellitus (2.6, 0.5); vntr (2.6, 0.5); mammals (2.6, 0.5); prone (2.6, 0.5) |
| 7 | 31 | 0.893 | 2008 | haplotype (10.67, 0.005); receptor (9.83, 0.005); african american (9.83, 0.005); disequilibrium (9.83, 0.005); linkage analysis (6.98, 0.01); risk (6.52, 0.05); body mas (6.19, 0.05); blood mononuclear cell (6.19, 0.05); type 2 diabetes (5.75, 0.05); type 2 diabetes susceptibility (4.91, 0.05); neuropsychiatric conditions (4.91, 0.05); serum creatinine (4.91, 0.05); e23k variant (4.91, 0.05); community (4.91, 0.05); undigested carbohydrates (4.91, 0.05); host response (4.91, 0.05); notch (4.91, 0.05); chromothripsis (4.91, 0.05); association analysis (4.91, 0.05); hnf1 alpha gene (4.91, 0.05); gene-environment interaction (4.91, 0.05); genes controlling variation (4.91, 0.05); secretion (4.91, 0.05); mars study (4.91, 0.05); p4 medicine (4.91, 0.05); interleukin 6 production (4.91, 0.05); chromosomal instability (4.91, 0.05); antipsychotic treatment (4.91, 0.05); apolipoprotein b gene (4.91, 0.05); psoriasis (4.91, 0.05); endoplasmic reticulum stress (4.91, 0.05); quebec family (4.91, 0.05); autonomic neuropathy (4.91, 0.05); molecular network (4.91, 0.05); powerful (4.91, 0.05); allostasis (4.91, 0.05); ets (4.91, 0.05); army (4.91, 0.05); sepsis (4.91, 0.05); lifestyle intervention (4.91, 0.05); neuroticism (4.91, 0.05); stimulation (4.91, 0.05); pi16 (4.91, 0.05); arginine methylation (4.91, 0.05); native american (4.91, 0.05); leucine repeat (4.91, 0.05); dyslipidemia (4.91, 0.05); wellness (4.91, 0.05); sleep disturbance (4.91, 0.05); anti-codon (4.91, 0.05); detect linkage (4.91, 0.05); large scale association (4.91, 0.05); baboon (4.91, 0.05); response prediction (4.91, 0.05); intensive care unit (4.91, 0.05); hiv 1 infection (4.91, 0.05); cell surface marker (4.91, 0.05); autoimmune disorders (4.91, 0.05); allostatic load (4.91, 0.05); genome-wide (4.91, 0.05); vegf (4.91, 0.05); posttranslational modification (4.91, 0.05); vein (4.91, 0.05); factor ngf (4.91, 0.05); artery (4.91, 0.05); genomic imprinting (4.91, 0.05); cardio-metabolic health (4.91, 0.05); dietary fiber (4.91, 0.05); weight gain (4.91, 0.05); 3go (4.91, 0.05); homeostasis model assessment (4.91, 0.05); chromoanagenesis (4.91, 0.05); test (4.91, 0.05); acetyltransferase complexe (4.91, 0.05); visceral obesity (4.91, 0.05); wide scan (4.91, 0.05); duration (4.91, 0.05); codon (4.91, 0.05); epigenetic repression (4.91, 0.05); peripheral blood mononuclear cell (4.91, 0.05); nonhuman primate (4.91, 0.05); polygenic risk score (4.91, 0.05); next-generation (4.91, 0.05); nerve growth factor (4.91, 0.05); critically ill (4.91, 0.05); healthspan (4.91, 0.05); complex genomic rearrangement (4.91, 0.05); kidney function (4.91, 0.05); kir6.2 (4.91, 0.05); proopiomelanocortin pomc gene (4.91, 0.05); pro12ala variant (4.91, 0.05); physical activity (4.91, 0.05); urinary albumin excretion (4.91, 0.05); symptom (4.91, 0.05); comparative genomic hybridization (4.91, 0.05); homeostasis (4.91, 0.05); type-1 diabetes (4.91, 0.05); regulatory t cells (4.91, 0.05); frequency (4.91, 0.05); serum leptin level (4.91, 0.05) |
| 8 | 28 | 0.923 | 2008 | diabetes (31.91, 1.0E-4); enterovirus (11.58, 0.001); whole genome shotgun (5.78, 0.05); enteroendocrine cell (5.78, 0.05); machine learning (5.78, 0.05); putative artificial selective sweeps (5.78, 0.05); glucose metabolism (5.78, 0.05); type ii diabetes mellitus (5.78, 0.05); glucagon-like peptide-1 (glp-1) (5.78, 0.05); pancreatic beta cells (5.78, 0.05); population genetic (5.78, 0.05); mody (5.78, 0.05); signal transduction (5.78, 0.05); nitric-oxide synthase (5.78, 0.05); repeated passages (5.78, 0.05); nutrient sensing (5.78, 0.05); c3h (5.78, 0.05); nucleotide sequence (5.78, 0.05); human genetics (5.78, 0.05); otsuka long evans tokushima fatty rat (5.78, 0.05); beta-cell function (5.78, 0.05); coronary (5.78, 0.05); 16s amplicon (5.78, 0.05); mouse pancreas (5.78, 0.05); zebrafish (5.78, 0.05); gk rat (5.78, 0.05); insulin action (5.78, 0.05); dyslipidaemia (5.78, 0.05); insulin-dependent diabetes mellitus (5.78, 0.05); dmo1 (5.78, 0.05); genetic determinants (5.78, 0.05); oriental herbal medicine (5.78, 0.05); sequence analyses (5.78, 0.05); genomics analysis (5.78, 0.05); citrus aurantium l. (ca) (5.78, 0.05); human skin (5.78, 0.05); angioplasty (5.78, 0.05); autoimmune (5.78, 0.05); endocrine cell differentiation (5.78, 0.05); down regulation (5.78, 0.05); gwas (5.78, 0.05); dog (5.78, 0.05); supervised learning (5.78, 0.05); exome (5.78, 0.05); coxsackievirus b4 (5.78, 0.05); chronic inflammation (5.78, 0.05); nkx6-1 (5.78, 0.05); increased blood glucose (5.78, 0.05); c57bl/6 (5.78, 0.05); immune (5.78, 0.05); regeneration (5.78, 0.05); coxsackievirus (5.78, 0.05); beta cell mass (5.78, 0.05); idd4 (5.78, 0.05); in stent restenosis (5.78, 0.05); glucose-stimulated insulin secretion (5.78, 0.05); pas kinase (5.78, 0.05); reprogramming (5.78, 0.05); implantation (5.78, 0.05); endocrine pancreas (5.78, 0.05); glucokinase (5.78, 0.05); epigenomics (5.19, 0.05); epigenetics (4.37, 0.05); beta cell (3.77, 0.1); association (3.2, 0.1); next generation sequencer (3.13, 0.1); epigenetic marks (3.13, 0.1); haplotypes (3.13, 0.1); genome scan (3.13, 0.1); genetic susceptibility (3.13, 0.1); histone acetylation (3.13, 0.1); muscle (3.13, 0.1); pancreatic islets (3.13, 0.1); candidate disease genes (3.13, 0.1); infection (3.13, 0.1); high throughput sequencing (3.13, 0.1); genetic analysis (3.13, 0.1); diet (3.13, 0.1); vascular cells (3.13, 0.1); personalized management (3.13, 0.1); histone methylation (3.13, 0.1); chip-seq (3.13, 0.1); faire-seq (3.13, 0.1); microbiome (3.13, 0.1); streptozotocin (3.13, 0.1); classification (3.13, 0.1); congenic (3.13, 0.1); histone deacetylase (3.13, 0.1); regulation (3.13, 0.1); legacy effect (3.13, 0.1); review (3.13, 0.1); metagenomics (3.13, 0.1); personalized care (3.13, 0.1); non-obese diabetic mouse (3.13, 0.1); genetics (2.5, 0.5); gene (2.27, 0.5); transcriptome (2.19, 0.5); pancreatic islet (2.19, 0.5); epigenome (2.19, 0.5); positional cloning (2.19, 0.5) |
| 9 | 28 | 0.91 | 2010 | loci (11.37, 0.001); caloric restriction (10.64, 0.005); biology (10.64, 0.005); risk factor (9.53, 0.005); obesity (8.53, 0.005); nutrition (6.96, 0.01); density lipoprotein cholesterol (5.38, 0.05); common multifactorial diseases (5.31, 0.05); direct-to consumer testing (5.31, 0.05); human diversity (5.31, 0.05); human adaptation (5.31, 0.05); next generation sequencing (5.31, 0.05); hyperresponsiveness (5.31, 0.05); acs (5.31, 0.05); differential methylation (5.31, 0.05); evolution (5.31, 0.05); angiogenesis (5.31, 0.05); leopard (5.31, 0.05); gene expression profile (5.31, 0.05); natural selection (5.31, 0.05); inbred mice (5.31, 0.05); dba/2 (5.31, 0.05); whole genome amplification (5.31, 0.05); rage gene (5.31, 0.05); strategy (5.31, 0.05); renal transplantation (5.31, 0.05); kidney disease (5.31, 0.05); sirt1 (5.31, 0.05); alms1 (5.31, 0.05); bone (5.31, 0.05); recipient (5.31, 0.05); lipoprotein(a) (5.31, 0.05); evolutionary adaptation (5.31, 0.05); standing variation (5.31, 0.05); bisulphite sequencing (5.31, 0.05); infectious disease (5.31, 0.05); autophagy (5.31, 0.05); intensive glycemic control (5.31, 0.05); proteases (5.31, 0.05); diseases prediction models (5.31, 0.05); c57bl/6j (5.31, 0.05); visceral adipose tissue (5.31, 0.05); srage (5.31, 0.05); graft los (5.31, 0.05); lower extremity amputation (5.31, 0.05); airway hyperreactivity (5.31, 0.05); gly82ser polymorphism (5.31, 0.05); circadian clock (5.31, 0.05); fear potentiated startle (5.31, 0.05); cyclosporine (5.31, 0.05); ldl/metabolism (5.31, 0.05); mycophenolic acid (5.31, 0.05); ethical (5.31, 0.05); estrogen (5.31, 0.05); low density lipoprotein (5.31, 0.05); all cause mortality (5.31, 0.05); legal and social issues (5.31, 0.05); felidae (5.31, 0.05); c reactive protein (5.31, 0.05); human genome (5.31, 0.05); microvascular complication (5.31, 0.05); hyperglycemia-hyperinsulinemia (5.31, 0.05); platelets (5.31, 0.05); scan (5.31, 0.05); insulin signaling (5.31, 0.05); progress (5.31, 0.05); tissue factor (5.31, 0.05); carnivorous diet (5.31, 0.05); pedigree (5.31, 0.05); glomerular filtration rate (5.31, 0.05); gender (5.31, 0.05); daphnia (5.31, 0.05); acute rejection (5.31, 0.05); de novo assembly (5.31, 0.05); link (5.31, 0.05); genetic counseling (5.31, 0.05); statins (5.31, 0.05); diabetes (4.37, 0.05); cardiovascular disease (3.58, 0.1); mouse (3.58, 0.1); susceptibility gene (3.58, 0.1); diabetes mellitus (2.69, 0.5); cardiac protection (2.69, 0.5); menarche (2.69, 0.5); whole genome (2.69, 0.5); age (2.69, 0.5); identifies multiple (2.69, 0.5); cigarette smoking (2.69, 0.5); carbonylation (2.69, 0.5); differentially methylated genes (2.69, 0.5); statin therapy (2.69, 0.5); strain (2.69, 0.5); go analysis (2.69, 0.5); comparative genomics (2.69, 0.5); aldehyde dehydrogenase 2 (2.69, 0.5); follow up (2.69, 0.5); ingenuity pathway analysis (2.69, 0.5); lin28b (2.69, 0.5); snp (2.69, 0.5); transcription factors (2.69, 0.5) |
